# Heterogeneity in Malignant Pleural Mesothelioma

**DOI:** 10.3390/ijms19061603

**Published:** 2018-05-30

**Authors:** Kathrin Oehl, Bart Vrugt, Isabelle Opitz, Mayura Meerang

**Affiliations:** 1 Department of Thoracic Surgery, University Hospital Zurich, 8091 Zürich, Switzerland; Isabelle.Schmitt-Opitz@usz.ch; 2 Institute of Pathology and Molecular Pathology, University Hospital Zürich, 8091 Zürich, Switzerland; Bart.Vrugt@usz.ch

**Keywords:** mesothelioma, inter-tumor heterogeneity, temporal intra-tumor heterogeneity, spatial intra-tumor heterogeneity, chemoresistance, cancer stem cells, targeted therapy

## Abstract

Despite advances in malignant pleural mesothelioma therapy, life expectancy of affected patients remains short. The limited efficiency of treatment options is mainly caused by inter- and intra-tumor heterogeneity of mesotheliomas. This diversity can be observed at the morphological and molecular levels. Molecular analyses reveal a high heterogeneity (i) between patients; (ii) within different areas of a given tumor in terms of different clonal compositions; and (iii) during treatment over time. The aim of the present review is to highlight this diversity and its therapeutic implications.

## 1. Introduction

Malignant Pleural Mesothelioma (MPM) is a rare and aggressive neoplasm arising from a layer of mesothelial cells lining the pleura. The main cause of MPM is exposure to asbestos fibers that provoke constant inflammation and malignant transformation of mesothelial cells by direct mitotic spindle interference, reactive oxygen species release, and macrophage attraction [1]. The latency of the cancer is about 40 years, but once diagnosed, the life expectancy without treatment is less than 12 months [2]. The treatment usually includes chemotherapy followed by surgery, which can prolong the median survival to 22 months [3]. However, the chemotherapy is only effective in approximately 30–40% of the patients [4]. In addition, an effective alternative treatment or second line treatment has not yet been established [5]. With the exception of a recent phase three trial combining Bevacizumab with the standard cisplatin and pemetrexed chemotherapy in newly diagnosed MPM [6], clinical trials aiming for a targeted therapy approach in common cancer signaling pathways have not resulted in a better overall survival (OS) [7]. These studies stress the need for new biomarkers to predict the clinical response to chemotherapy as well as to find new possible targets for alternative therapy approaches. The search for new treatment options is complicated by the genetic composition of the tumor. Mutations are mainly found in tumor suppressors (COSMIC [8]), but common oncogenes such as *PI3K*, *EGFR*, and *VEGFR* are, if any, rarely found to be mutated in MPM, which limits the choice of targeted inhibitors. Although studies have shown that loss of tumor suppressors, such as *NF2* and *CDKN2A/p16,* lead to upregulation of associated oncogenic pathways, the translation of this knowledge into effective treatments has not yet occurred.

The mechanisms underlying the poor response of patients with MPM to a wide range of therapeutic interventions is still unknown. One reason for the inefficacy of the treatment regimens is the molecular inter-tumor heterogeneity, describing the diverse mutational (referred to as “genetic” in this review), epigenetic, expressional, and macroscropic (summarized as “phenotypical”) changes between patients. Many mutations, such as in *EGFR* or *TP53*, are undetectable in the majority of MPM cases (COSMIC [8]). In contrast to non-small cell lung cancer [9], the relatively low number of MPM cases combined with the low prevalence of drug-targetable EGFR mutations in MPM compromises the investigation and use of selective EGFR inhibitors in the treatment of mesothelioma.

Adding to the complexity that arises due to inter-tumor heterogeneity, patient tumors also display intra-tumor heterogeneity. The existence of several tumor clones and subclones within one tumor sample of the same patient significantly limits the ability to devise logical treatment strategies. Intra-tumor heterogeneity appears during the course of the disease (temporal intra-tumor heterogeneity) as well as in different locations within the tumor at one time point (spatial intra-tumor heterogeneity).

Histologically, temporal and spatial intra-tumor heterogeneity in MPM manifests with a morphological spectrum, ranging from epithelioid to sarcomatoid tumors with the biphasic subtype containing a combination of both epithelioid and sarcomatoid components, each constituting at least 10% of the tumor. Adding to the complexity of histological subtyping, morphological biomarkers in epithelioid MPM, including nuclear atypia and number of mitoses, have been used to determine a total score which independently correlates with overall survival [10]. This further supports the existence of tumor heterogeneity, even within morphological well-defined subgroups of MPM. Furthermore some MPMs show a change of histology during the course of the disease, which represents temporal heterogeneity [11]. Besides this microscopic diversity, an increasing number of publications highlight the importance of genetic intra-tumor heterogeneity for therapeutic resistances in several cancer types [12]. Until now, this phenomenon has attracted little attention in MPM. 

The aim of the present review is to highlight the different forms of heterogeneity in MPM with emphasis on the genetic and phenotypic intra-tumor heterogeneity. We summarize evidence of the spatial and temporal evolution of MPM, during the treatment with standard of care chemotherapy, and discuss the implications of heterogeneity on treatment decisions.

## 2. Inter-Tumor Heterogeneity

MPMs are known to have a high degree of molecular inter-tumor heterogeneity. In terms of genetic alterations, MPM generally displays a low number of mutations and recurrent mutations compared to other cancers [13]. The genes that were reported to be most often mutated are *BAP1* and *NF2*. Other commonly detected SNVs are found in *LATS1/2*, *TP53*, and *TERT* [14,15]. More prominent than SNVs are large chromosomal aberrations, which are thought to arise from direct interference with asbestos fibers or general chromosomal instability due to dysfunctional DNA damage response [1]. Chromosomal losses are the most frequent alterations in MPM, mostly affecting the chromosomal arms 3p, 9p, and 22q, where, amongst others genes, *BAP1*, *CDKN2A*, and *NF2* are located, respectively [8,16,17]. A high number of patients even harbor homozygous deletions of the *CDKN2A* region [18].

Despite these common alterations, the composition and gene locations of the mutations vary considerably between patients. A large sequencing study by Lo Iacono and colleagues, using 123 FFPE samples, sequenced 50 genes using the AmpliSeq Cancer Hotspot Panel plus another custom-designed amplicon panel covering the exons of the *NF2* and *BAP1* genes [19]. Although the authors reported a higher number of mutations clustering in exon 13 and 17 of the *BAP1* gene, which are the two largest exons, it did not seem that those were common hotspots for *BAP1* mutations (COSMIC [8]); there was more of an enrichment found in the N-terminal Ubiquitin Hydrolase domain (COSMIC [8]). Another study by Guo et al. compared 22 MPM tumor samples with matched blood samples using exome sequencing [13]. In total, they detected 490 somatic protein-altering mutations of which 477 were private alterations. Another working group led by Mäki-Nevala also performed exome-sequencing on 21 patients (two of them with peritoneal mesothelioma) and only found two non-private mutations in TTLL6 and MRPL1 occurring in two asbestos-exposed MM patients [20]. Ugurluer and colleagues as well as Kato and colleagues [21,22] both used a large gene panel covering 236 genes. Both groups, analyzing 11 [21] and 42 [22] mesothelioma patients, also failed to find any non-private alterations. Other groups working with smaller gene panels [14,23] also showed only private mutations. The results from these publications clearly illustrate that, in contrast to e.g., the L858R mutation in EGFR in lung cancer [24], there are no commonly mutated amino acid positions or “hotspot” regions in any of the genes tested.

In summary, these molecular analyses highlight the high inter-patient variability of locations and compositions of mutational patterns. This heterogeneity compromises the use of targeted therapy for mesothelioma patients and necessitates a personalized approach (Table 1). Clinical trials inhibiting for example the EGFR receptor in MPM patients using Erlotinib (NCT01592383, NCT00137826, NCT00039182), Gefitinib (NCT00787410, NCT00025207), Vandetanib (NCT00597116), or Cetuximab (NCT00996567) did not reveal any beneficial effects of the treatment. Although the mutational rate of EGFR is below 1% in MPM (COSMIC [8]), the rationale of those studies were the overexpression of EGFR which is found in over 50% of cases [25,26]. Destro and colleagues stained tumor tissue of 61 patients, whereby positive staining in 0–10% of tumor cells was regarded as negative expression, in 10–50% as low, and in >50% as high [25]. Only 9/61 (14.8%) showed a high EGFR expression, whereas 41.0% (21/61) only showed a staining in less than 50% of tumor cells, indicating that only a subpopulation of tumor cells overexpress EGFR. Enomoto and colleagues also stained 22 MPM cases, setting the thresholds for score 1+ for <5% positive tumor cells, score 2+ for 5–50% and score 3+ for >50% [26]. They scored 50% (11/22) of tumors as 3+ expression. Based on the assumption that high EGFR expression predicts the success of EGFR inhibiting drugs such as Erlotinib, detection of strong positive staining should be used as inclusion criteria in future studies. However, it was already shown that in many cancers, EGFR expression levels are not associated with a positive response to targeted therapy [27]. This was also documented in MPM by Garland and colleagues assessing EGFR expression in 57 patients with MPM [28]. A score of 0 was given for negative staining, score 1 for weak and focal staining, score 2 for positive and homogenous staining and score 3 for intense staining. In their cohort, 75% of the tumors stained score 2 or 3 for EGFR. However, no objective clinical responses to Erlotinib treatment was noted. Similar results were shown using Gefitinib [29] and Cetuximab [30], which strongly indicates that high EGFR expression cannot be used to predict response to EGFR inhibitors in patients with mesothelioma.

In our sequencing studies (Oehl et al., manuscript in preparation), we could see *EGFR* mutations at low allele frequency in the tissues, indicating a subclonal origin. Further, EGFR staining, as described above, often shows a focal pattern. Both findings suggest that there could be, additionally to the inter-tumor variability, a high intra-tumor heterogeneity additively influencing the outcome of anti-EGFR treatments in a negative way.

## 3. Spatial Intra-Tumor Heterogeneity

### 3.1. Spatial Genetic Heterogeneity

MPM is known to show intrinsic therapy resistances and is so far non-curable. The high number of non-responders to chemotherapy [4] as well as the frequent recurrences of the disease [37,38] suggest a substantial degree of resistant clones within an MPM patient. In silico modeling of spatial tumor growth suggests that the number of driver gene mutations, as well as the speed of cell turnover, greatly influences the degree of heterogeneity within a tumor [39]. Interestingly, the model proposed by Waclaw et al. shows that fewer driver mutations and a slow cell turnover lead to an increased level of heterogeneity [39]. Given that mesothelioma is supposed to develop over many years, the replication rate is in most cases quite low, indicating that there should be a very high degree of molecular diversity within the tumor.

Indeed, Comertpay and colleagues assessed the clonality of malignant mesothelioma in 14 female patients using a HUMARA assay [40]. This assay is based on X-chromosome inactivation by methylation and the *HUMARA* gene which is located on the X-chromosome. This gene encodes for the Human Androgen Receptor and harbors a varying number of CAG repeats, which usually differs between the maternal and paternal allele. One allele gets deactivated in healthy females; therefore, if a cancer was of monoclonal origin, only one allele would be detected in the tumor. However, when using the HUMARA assay on MPM tissue, Comertpay et al. detected paternal and maternal *HUMARA* alleles within most of the tumors, indicative of a polyclonal origin of MPM.

As described above, a common molecular alteration is the homozygous deletion of *CDKN2A* (p16) on chromosome 9. However, when measured by fluorescent in-situ hybridization (FISH) on tumor tissue, it is well known that the homozygous deletion cannot be detected in all cells of the tumor. Indeed, the status of the *CDKN2A* gene is highly variable, with no detectable loss, hemizygous losses and homozygous losses of *CDKN2A* within the same tumor. Defining a tumor as “homozygously deleted for *CDKN2A*” therefore requires defined cut-offs, such as 14.4% in a study by Wu et al. that compared the homozygous deletion patterns of *CDKN2A* between sarcomatoid mesothelioma and fibrous pleuritis [41]. These detections of non-homogenous deletions of *CDKN2A* suggest that besides the polyclonal origin, several genetic subclones might also exist within one tumor.

However, the only study so far describing genetic spatial heterogeneity was recently conducted by Kiyotani and colleagues [42]. From the surgical specimens of six MPM patients, they extracted DNA and RNA from fresh frozen tissue from three different locations within the tumor, namely from anterior, posterior, and diaphragm positions. They then conducted whole-exome sequencing, resulting in 19–47 non-synonymous mutations per sample. When looking at the SNVs that were detected at the three different locations within one patient, they found clearly distinct mutational patterns. Comparing the allele frequencies of these mutations, they detected some high variant allele frequency mutations in every examined location of the respective tumor, indicative of mutations of early clonal origin. Moreover, they saw a high degree of intra-tumoral spatial heterogeneity represented by varying amounts of subclonal fractions. The addition of TCRβ sequencing data and immune-related gene expression analysis revealed that this heterogeneity also extends to the immune microenvironment.

### 3.2. Spatial Phenotypic and Tumor Microenvironment Heterogeneity

**Figure 1:**
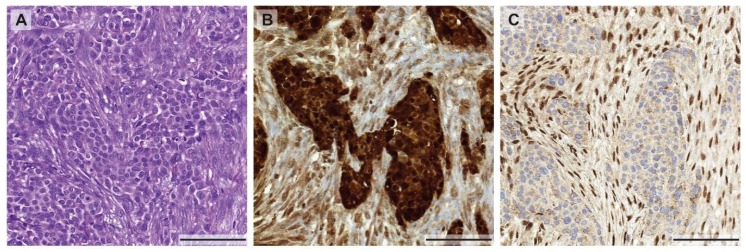
Morphological and immunohistochemical heterogeneity in mesothelioma. (**A**) Biphasic mesothelioma consisting of an epithelioid and sarcomatoid component (H&E stain), highlighted by a calretinin staining (**B**) showing a weaker expression in the sarcomatoid proliferation. (**C**) Heterogeneous expression of BAP-1 with positive nuclear staining in the sarcomatoid component and loss of BAP-1 expression in the epithelioid areas of the tumor. All pictures were taken at 10× magnification (scale bar: 100 µm).

As mentioned above, tumor heterogeneity is not only described by a heterogeneous genetic makeup of tumor cells within the same patient. The heterogeneity can also arise from selective environmental pressure such as nutrient, oxygen, tumor stroma, and immune microenvironment that can induce tumor heterogeneity by altering their phenotypes. This selective pressure of the microenvironment can govern the tumor phenotype by altering signaling pathways, regulating gene and protein expression. These intra-tumoral differences in the environment could result in therapy resistances [43]. To support this idea, it has been clearly demonstrated that hypoxic tumors are more resistant to chemotherapy and radiotherapy [44]. A recent study visualized tumor hypoxia by non-invasive imaging, [F-18] fluoromisonidazole (FMISO) PET-CT, and demonstrated that MPM has a visible area of hypoxia, predominantly in bulky tumor masses [45]. Thus, tumor cells in different regions of the tumor nodule may respond differentially to treatment. In view of immunotherapeutic approaches, for example using PD-1 or PD-L1 blocking antibodies, the heterogeneity of PD-L1 protein levels between primary and metastatic sites was recently studied in 64 MPM patients [46]. It was shown that PD-L1 expression, measured by immunohistochemical staining, was discordant in up to 31% of the cases (depending on the reviewer), which pronounces the limits of successful immunotherapy using anti-PD-L1 antibodies in mesothelioma. However, as seen in the example of the focal EGFR staining described above, the heterogeneity of protein expression is not only found in primary tumors and metastases but it also occurs within different regions within the same primary tumor. For example, loss of BAP1 expression in biphasic MPM can only be found in the epithelioid part of the tumor (Figure 1), whereas BAP1 is retained in the sarcomatoid component. The different expression profiles in different parts of the tumor could be related to the genetic or epigenetic background and require further investigation.

Although some studies indicate that genetic, phenotypic, and microenvironmental intra-tumor heterogeneity in MPM exist, our knowledge is still limited. More studies assessing spatial MPM heterogeneity are needed to improve our understanding of the pathophysiological mechanisms underlying MPM and to develop new treatment approaches that circumvent the impact of intra-tumor heterogeneity.

**Figure 2:**
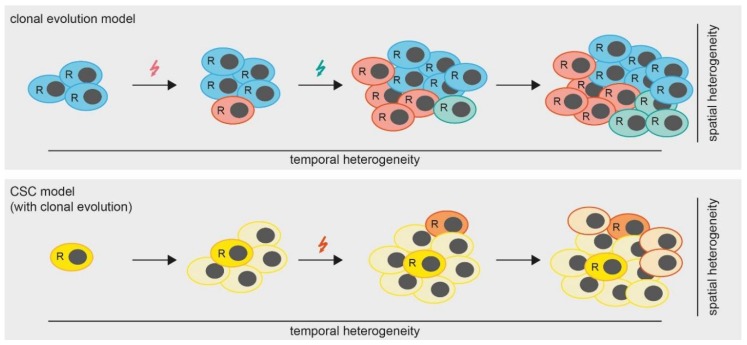
Models of tumor heterogeneity. In the clonal evolution model (upper panel) all cells are able to replicate (indicated by “R”). Mutations (colored arrows) are gained over time, leading to the formation of subclones which results in a heterogeneous tumor. In the cancer stem cell (CSC) model with clonal evolution, only CSCs are able to replicate. However, mutations occurring over time lead to the formation of additional CSCs.

However, the question remains, which mechanisms lead to the development of spatial heterogeneity in MPM? A widely accepted theory (besides the cancer stem cell (CSC) theory which we will discuss later in this review) is that of clonal tumor evolution [47]. Hereby, a tumor accumulates somatic mutations and chromosomal aberrations in a stepwise manner. Some of these alterations are so-called “driver mutations”, conferring a fitness advantage to the respective clones and leading to the development of several subclones, which are ultimately detected as spatial heterogeneity (Figure 2). Therefore, the spatial heterogeneity within a tumor can be seen as the result of a temporal heterogeneity, which we will discuss in the next chapters.

## 4. Temporal Intra-Tumor Heterogeneity

Mesothelioma is often diagnosed at an advanced stage due to late onset and non-specific symptoms. Surgery and cytotoxic chemotherapy, platinum (*cis*- or carboplatin) plus pemetrexed, are standard first-line treatment for patients with MPM. Nevertheless, the prognosis of MPM remains poor because of tumor recurrence within a median time of 10–18 months after initial treatment [2]. 

As described above, tumors can evolve over time during multiple rounds of cell division. The presence of selective pressure such as treatment with an anti-cancer drug is an additional factor driving tumor clonal evolution (Figure 2). This temporal heterogeneity has severe implications for treatment decisions, as seen in various cancer entities. For example, a study in medulloblastoma, revealed that genetic aberrations of recurrent tumor tissues following the treatment with chemotherapy and radiotherapy diverged from that of diagnostic (treatment naive) tissues. This was due to the selection of the preexisting subclones that were already present before the treatment [48]. Another study in breast cancer observed an enrichment of slow proliferating cell populations with different molecular and biological characteristics following the treatment with chemotherapy, depending on the subtype of cancer. Employing single cell FISH analysis, they further demonstrated that breast cancer patients with low genetic heterogeneity responded better to the treatment [49]. To date, there is no study directly assessing temporal MPM heterogeneity and its implication on treatment outcomes.

### 4.1. Chemotherapy and Tumor Heterogeneity 

Cisplatin and pemetrexed are the common cytotoxic agents given to MPM patients as a first-line treatment. Cisplatin is a genotoxic drug that induces intrastrand DNA cross-linking, inducing DNA damage, growth arrest, and cell death [50]. Pemetrexed is a folate antimetabolite that inhibits three enzymes involved in purine and pyrimidine synthesis. The lack of purine and pyrimidine results in the inhibition of deoxyribonucleic acid (DNA) and ribonucleic acid (RNA) synthesis, essential elements for cell proliferation and survival. Treatment with cisplatin-pemetrexed may eradicate sensitive and highly proliferative cells, but the resistant cells remain and can still grow or regrow following the treatment. Thus, this selective pressure can alter the composition of the tumor cell population and the extent of tumor heterogeneity following the treatment. Moreover, these cytotoxic agents can change the biological characteristics of tumor cells. For example, changes in the expression of genes associated with both cellular senescence (*PAI-1* and *IL-6*) and a gene identified as a cancer stem cell marker (*Thy-1*) were detected in primary MPM cells following exposure to cisplatin-pemetrexed. Moreover, increased Thy-1 expression was observed in acquired cisplatin pemetrexed resistant cells in vitro [51]. In addition to inducing phenotypical changes, DNA damage caused by cisplatin, if incorrectly repaired, can generate novel mutations or chromosome alterations, thereby increasing genetic diversity of tumors. Nonetheless, chemotherapy may generate a novel targetable mutation or cause an enrichment of targetable mutations that are not detectable prior to the treatment.

Thus, knowing the genetic and phenotypical changes of relapse tumors can be useful for the selection of effective second line treatment. For MPM, there has been no study assessing the effect of chemotherapy on the temporal heterogeneity. To study whether the genetic makeup of MPM changes over the course of treatment and disease progression, we compare the mutation profile of MPM in tumor tissues collected at three different time points during treatment (pre-chemo, post-chemo, and recurrence). Preliminary data (Oehl et al., manuscript in preparation), suggests that the genetic basis of some of the MPM tumors change over the course of treatment with chemotherapy.

Phenotypic changes of MPM tumor cells following the treatment with chemotherapy have also been observed in some studies. Indeed, the histologic subtype of the tumor changes over the course of therapy [11]. We also observed protein expression changes of MPM tumors following the treatment with chemotherapy. In our previous study [52], we compared protein expression of markers of the PI3K-mTOR pathway namely PTEN, p-mTOR, and p-S6 in matched MPM tissues pre- and post- cisplatin-based induction chemotherapy. Staining of the tissue microarray (TMA) revealed a reduction of protein expression of PTEN, p-mTOR, and p-S6 in the tumor tissues following chemotherapy. A decrease in tumor proliferative activity (Ki-67 expression) and slightly increased numbers of apoptotic cells (cleaved Caspase-3 staining) were also detected following the treatment. In another study [53] using the same patient cohort, we observed increased NF2 (Merlin) expression and decreased Survivin labelling in post chemotherapy treatment tissues (unpublished data). Employing a cohort of 34 patients with pre- and post- chemotherapeutic tissues available, Sidi et al. demonstrated that expression of senescence marker genes such as *PAI-1* and *p21* was significantly increased after chemotherapy [54].

These studies show that sampling at different time points during MPM treatment might reveal new potential treatment targets, which were not detectable at the time of diagnosis. Thus, longitudinal analysis of tumor tissues may be useful for the selection of subsequent effective therapies for MPM patients.

### 4.2. MPM Cancer Stem Cell

Cancer stem cells also play a role in tumor heterogeneity. Cancer stem cells represent a small population of tumor cells that are able to self-renew (Figure 2). Upon cell division, cancer stem cells give rise to progeny cells that maintain self-renewal properties or differentiate into various cell entities. It is widely demonstrated that cancer stem cells are commonly resistant to various anti-cancer drugs. Although the stem cell model remains controversial, cells with stem-cell like properties have been shown to contribute to tumor heterogeneity and therapy resistance in many solid tumors [55].

Relying on the basis that cancer stem cells express high levels of membrane drug transporter ABCG2, a study by Kai et al., employed Hoechst 33342 dye efflux assay to identify a MPM stem cell population [56]. Using this assay, they detected a small subset of cells (side population; SP) that can exclude Hoechst dye in three MPM cell lines and a transformed mesothelial cell line (MeT-5A) (number ranging from 0.05–1.32%). Treatment with cisplatin substantially increased the SP fraction of MPM cells. Interestingly, this SP population expressed higher levels of stem cell related genes namely *BMI1*, *OCT4*, and *NOTCH1* compared to non-SP (NSP) cells. However, despite exhibiting enhanced proliferation in vitro, there was no difference in in vivo tumorigenicity of both SP and NSP when implanted subcutaneously in NOD/SCID mice. Another study by Frei et al., also employed the same functional assay (using DyeCycleViolet) to identify SP on MPM cell lines and primary cells [57]. This study could also detect a small population of SP in all cell lines tested (ranging from 0.2–1%). Similar to the previous study, there was no difference in tumorigenicity between SP and NSP when implanted under the renal capsular of NOD/SCID mice. However, when SP cells were sorted from the in vivo tumor tissues, these tended to be more tumorigenic compared to NSP (although this was not statistically significant). MPM SP were however more resistant to cisplatin and expressed increased level of *PTCH1*, a gene of the sonic hedgehog signaling pathway.

Kim Chul et al. further characterized MPM SP using genome wide DNA methylation profiling coupled with mRNA expression [58]. This study described increased DNA methylation in CpG islands, gene flanking, and intragenic regions in SP cells compared to NSP. They identified 1130 genes differentially expressed in SP compared to NSP, among which, 122 genes are known to be regulated by aberrant DNA methylation. Importantly, these candidate genes, such as *YAP1* and *NOTCH2*, are known to play an important role in the maintenance of stem cell and the regulation of differentiation and development.

High level of ALDH1A has been used as a marker of CSCs. Thus, a study by Shapiro, et al. identified a CSC population of Merlin (NF2) negative MPM cell lines using the Aldefluor assay [59]. Here, they detected MPM CSCs (Aldefluor + cells) with increased tumor initiating potential compared to non-CSCs when implanted into immunodeficient mice. Treatment with cisplatin-pemetrexed increased this CSC population of MPM cell lines while treatment with the FAK inhibitor VS-4718 reduced the number of CSCs in vitro. Using a preclinical patient derived xenograft (PDX) model, they further demonstrated that treatment with the FAK inhibitor targeting CSC populations was effective in the control of tumor growth following cisplatin-pemetrexed treatment, that had enriched the CSC population.

In conclusion, SP displaying CSC characteristics have been identified in MPM cell lines and primary cells. In a preclinical model, CSCs survive during the treatment with cisplatin and pemetrexed and thus can give rise to a new generation of tumor cell population and create the diversity of recurrent tumor clones that differ from tumor at diagnosis. Thus far, all studies isolated potential MPM CSCs from cell lines, thus more evidence on the existence of CSCs in MPM clinical specimens and their role in treatment resistant is needed. An important factor when investigating CSCs in MPM is to take into account the intrinsic self-renewing capacity of mesothelial tissues which is conferred by mesothelial progenitor cells [60]. Given the heterogeneous and polyclonal nature of MPM, one can speculate that there exist several MPM CSC clones with different genetic alterations. These can also stem from clonal evolution of CSCs that acquire mutations over time during disease progression (Figure 2). This scenario would further increase the complexity of MPM heterogeneity.

## 5. Implications for Therapy

Results from clinical trials testing targeted treatments in MPM have so far been discouraging. As illustrated above, one factor responsible for poor treatment outcome is inter-patient variability in the expression or mutational status of the target molecules. Thus, appropriate predictive markers for targeted treatments are needed for the design and implementation of clinical studies. Although stratification of patients regarding to predictive markers is difficult to realize given the low incidence of MPM, this personalized treatment is probably the only way to overcome the high inter-patient heterogeneity of the disease.

As discussed above, another level of heterogeneity, namely the intra-tumor heterogeneity observed on both the genetic and phenotypic level in MPM, is another hurdle for the success of MPM treatment. MPM tumor may comprise of heterogeneous variants of tumor cells possessing different levels of chemosensitivity that can affect macroscopic response outcomes. The remaining chemoresistant cells can progress and reestablish tumor heterogeneity in the relapse tumor. Cancer stem cells may also play a role in chemoresistance of MPM. Their role in actual tumor and disease progression, however, remains to be elucidated. If CSCs can be defined in the patient tumor tissue, they might represent a potent target for therapy approaches to overcome chemotherapy resistance of MPM. Intra-tumor heterogeneity is also a major factor that complicates the development of new targeted agents. Treatment targeting only a small subpopulation of tumor will not be effective, but targeting the core gene or pathway alteration that are shared across all tumor cells will provide the most efficient way to eradicate tumors. To address genetic heterogeneity, an obvious approach would be to model the tumor evolution by either single cell or tumor bulk sequencing to define driving mutations of early clonal origin. Understanding the pathways behind those mutations could then be exploited to develop new therapeutic approaches targeting most, if not all, of the tumor cells. However, using this approach it will be inevitable to monitor the development of the tumor over the course of treatment, since the probability of selecting resistant clones is quite high, as seen previously for example in lung adenocarcinoma [61]. This monitoring will further provide important information for the selection of subsequent effective treatment.

In general, the tracking and multi-level analysis of the tumor tissue implies the need for sufficient material. This is usually available after surgery (if conducted), but in order to reveal alternative treatment options prior to or instead of operative interventions and chemotherapy, it will be necessary to analyze the tissue derived from the diagnostic biopsy. Therefore, it is critical to remove an adequate amount of tissue.

Furthermore, knowing that MPM is a heterogeneous disease, single-region sampling is unlikely to reflect the complete genetic and phenotypic landscape of the tumor. Therefore, multi-region sampling is required in order to determine dominant clones and potential therapy resistant subclones.

Since this approach might be difficult to be carried out in clinics, an alternative option could be the detection of circulating cell free tumor DNA (ctDNA) in the blood or pleural fluid of MPM patients [62]. In this scenario, ctDNA is a pool of DNA released from tumor cells at different locations thus could serve as a good representation of genomic heterogeneity. A recent study in colorectal cancer for example collected plasma samples at different time points during multimodality treatment [63]. They could show that patients that tested mutation-positive in their ctDNA after chemoradiotherapy or surgery had an increased risk of recurrence. A previous study by the same group, also in colorectal cancer, could even show that the median lead time between ctDNA detection and radiological recurrence was higher than five months [64]. However, both studies relied on the detection of mutations that were previously found in the primary cancers of the respective patients, and thus could not identify novel mutations or mutations that were enriched during the treatment course. In order to address this drawback, other groups like Shu et al. used a panel of 382 cancer-associated genes for sequencing of ctDNA from various cancer entities and reported several mutations that were not found in the corresponding primary tumor, probably representing temporal or spatial heterogeneity [65]. A similar approach using MPM associated genes could be envisioned for the future monitoring of mesothelioma treatment. New next generation sequencing techniques and improved bioinformatical pipelines hereby enable the reliable detection of low frequency alleles and possible subclones also in clinical settings [66].

Taken together, intra-tumor heterogeneity and its importance in the treatment of cancer has been clearly demonstrated in recent years. It is predictable that MPM is a heterogeneous tumor as it takes 30–40 years from asbestos exposure to disease development with the tumor developing on a large surface of the mesothelial layer lining the thoracic cavity. Nevertheless, there has only been a limited number of publications addressing the intra-tumor heterogeneity of MPM, an aggressive malignancy with limited therapeutic options. Thus, more studies are required to dissect different levels of MPM intra-tumor heterogeneity at different time points during treatment. Clearly, a better understanding of MPM evolution is essential for designing more effective treatment regimens.

## Figures and Tables

**Table 1:** Selection of finished studies using targeted therapy approaches in malignant pleural mesothelioma (MPM). The mutational rate in MPM was taken from the cosmic database. Data on expression were taken from indicated references.

Target	Drug	Study	Year of Completion	Status	# Patients	Results	Marker	Mutational Rate in MPM	Expression in MPM
mTor	Everolimus	NCT00770120	2014	completed	61	primary endpoint not reached	NF2 (Merlin)	17% (105/629)	4% [31]–8% [32] negative
		NCT01024946	2012	completed	11	none published			
FAK	Defactinib	NCT01870609	2016	terminated	344	lack of efficiency			
ALK1	PF-03446962	NCT01486368	2015	completed	17	primary endpoint not reached	ALK	0% (1/343)	0% [33]–20% [34] positive
EGFR	Erlotinib	NCT00039182	2007	completed	55	primary endpoints not reached	EGFR	1% (8/652)	15% [25], 50% [26], 75% [28] high
	Cetuximab	NCT00996567	2015	completed	22	primary endpoint not reached			
c-Met	Tivantinib	NCT01861301	2015	terminated	18	lack of efficiency	MET	1% (3/448)	17% [35]–40% [36] high

Background color highlights groups of studies that employed the same marker for patient stratification.
